# Predictors of round window accessibility for adult cochlear implantation based on pre-operative CT scan: a prospective observational study

**DOI:** 10.1186/s40463-015-0073-7

**Published:** 2015-05-28

**Authors:** Edward Park, Hosam Amoodi, Jafri Kuthubutheen, Joseph M. Chen, Julian M. Nedzelski, Vincent Y. W. Lin

**Affiliations:** Department of Otolaryngology – Head and Neck Surgery, Sunnybrook Health Sciences Centre, 2075 Bayview Avenue, Toronto, ON M4N 3M5 Canada

**Keywords:** Round window, Cochlear implant, CT scan

## Abstract

**Background:**

Cochlear implantation has become a mainstream treatment option for patients with severe to profound sensorineural hearing loss. During cochlear implant, there are key surgical steps which are influenced by anatomical variations between each patient. The aim of this study is to determine if there are potential predictors of difficulties that may be encountered during the cortical mastoidectomy, facial recess approach and round window access in cochlear implant surgery based upon pre-operative temporal bone CT scan.

**Methods:**

Fifty seven patients undergoing unilateral cochlear implantation were analyzed. Difficulty with 1) cortical mastoidectomy, 2) facial recess approach, and 3) round window access were scored intra-operatively by the surgeon in a blinded fashion (1 = “easy”, 2 = “moderate”, 3 = “difficult”). Pre-operative temporal bone CT scans were analyzed for 1) degree of mastoid aeration; 2) location of the sigmoid sinus; 3) height of the tegmen; 4) the presence of air cells in the facial recess, and 5) degree of round window bony overhang.

**Results:**

Poor mastoid aeration and lower tegmen position, but not the location of sigmoid sinus, are associated with greater difficulty with the cortical mastoidectomy. Presence of an air cell around the facial nerve was predictive of easier facial recess access. However, the degree of round window bony overhang was not predictive of difficulty associated with round window access.

**Conclusion:**

Certain parameters on the pre-operative temporal bone CT scan may be useful in predicting potential difficulties encountered during the key steps involved in cochlear implant surgery.

## Background

Cochlear implantation has become a widely accepted treatment option for patients with severe to profound sensorineural hearing loss. The benefits to the patient are well published in both pediatric and adult populations. Historically, the cochlear implant electrode was inserted through a cochleostomy, typically anterior-inferior to the presumed location of the round window. Currently, many large cochlear implant centres, including our own, have chosen the round window approach for the majority of electrode insertions. This was made possible mainly by the development of slimmer, atraumatic electrodes and through the popularization of the concept of “soft” hearing preservation surgical techniques [1].

There are several key surgical steps for a cochlear implant with the intention of a round window insertion. They include 1) cortical mastoidectomy; 2) opening the facial recess; and 3) round window membrane identification and opening. A cortical mastoidectomy is defined as a canal-wall-up mastoidectomy in which its main purpose is to establish the location of the mastoid antrum and allow access to the facial recess. The facial recess, also known as a posterior tympanotomy, is a well-established otologic surgical pathway that gains access to the middle ear without violating the tympanic membrane. Its borders are defined as the vertical segment of the facial nerve medially, the chorda tympanic nerve/tympanic annulus laterally and the incus buttress superiorly. This narrow, bony 3-dimensional space which comprises the facial recess can often be challenging to identify and expose in order to gain access to the round window located more posteriorly. Finally, the round window is usually partially hidden by the bony round window niche and this familiar landmark must be identified before the bony niche can be drilled away to fully expose the round window membrane. Once the round window membrane is fully exposed, then it can be opened to enter the perilymphatic space of the scala tympani before the electrode can be carefully and slowly inserted.

These well-established steps of cochlear implantation may be influenced by anatomical variations among patients, which can pose unanticipated technical challenges with respect to obtaining adequate surgical exposure. A pre-operative temporal bone CT scan, done routinely in many centres including ours, serves as a guide to the anatomical layout of the ear to be implanted. Our hypothesis is that by analyzing the pre-operative temporal bone CT scan, it may be possible to determine certain radiological features that can predict the level of difficulty with the aforementioned surgical steps. In turn, such information can help surgical trainees anticipate and prepare for technical challenges that may be encountered during the operation.

There are several previous studies that have assessed the relationship between the findings from pre-operative temporal bone CT scan and intraoperative findings of structural abnormalities during cochlear implant [2–4]. However, most of these studies have focused on cochlear patency/ossification and did not attempt to correlate intraoperative difficulties with pre-operative CT parameters. In the study by Woolley et al. [4], pre-operative CT findings were compared to intraoperative findings during pediatric cochlear implantation in a retrospective fashion, but there was no intraoperative grading to ‘quantify’ the difficulties associated with pertinent steps; instead, they described the difficulties and any intraoperative complications that occurred. In comparison, our study is a prospective study, which assessed the correlations between specific and easy-to-measure parameters on the pre-operative temporal bone CT and intraoperative difficulties with key surgical steps that were graded by the surgeon during cochlear implantation.

## Methods

### Study design

This was a prospective, observational study of consecutive cochlear implant surgeries with the goal of a round window insertion performed at an adult tertiary implant centre. All surgeries were performed by three surgeons who routinely perform round window electrode insertions. Patients with previous mastoid surgery, re-implantations, revision surgeries, and patients who were implanted via alternative techniques (e.g. transcanal) were excluded from the study.

### Ethics, consent, and permissions

The study was approved by the ethics board at the Sunnybrook Health Sciences Centre and the consent for participation in research study was taken as part of the consent for cochlear implant surgery.

### Subjects

A total of 57 patients who underwent unilateral cochlear implant with round window electrode insertion and had pre-operative high-resolution temporal bone CT scan were included. All patients had moderate to profound bilateral sensorineural hearing loss, which was unaidable with hearing aids. All patients underwent routine audiometric testing and pre-operative electronystagmography, as well as temporal bone CT scans. They also received pre-operative counseling from a cochlear implant audiologist as part of our screening protocol.

### Intra-operative scoring of surgical difficulties

Difficulties encountered with each of the three key intraoperative steps - cortical mastoidectomy, access to the facial recess, and round window access - were scored according to the following scale: 1 = “easy”, 2 = “moderate”, 3 = “difficult”. Scoring was performed by the primary surgeon, who was a staff otologist, or by the fully credentialed otology fellow. They were blinded to the potential predictors of difficulties with the aforementioned surgical steps on the pre-operative CT scan.

### Analysis of pre-operative CT scan

Pre-operative temporal bone CT scans were analyzed by the primary author, who was blinded to the intra-operative scoring of surgical difficulties. The CT scan contained high-resolution images that are 0.625 mm thick with reconstructed coronal and sagittal images. The images were viewed in the standard bone window setting. The CT scan was analyzed for 1) degree of aeration of the mastoid; 2) location of the sigmoid sinus; 3) height of the tegmen, which correspond to difficulties associated with a cortical mastoidectomy, as well as 4) the presence of air cells around the facial recess, which relates to difficulty associated with performing the facial recess, and 5) degree of round window bony overhang, which relates to difficulties associated with round window access.

**Fig. 1:**
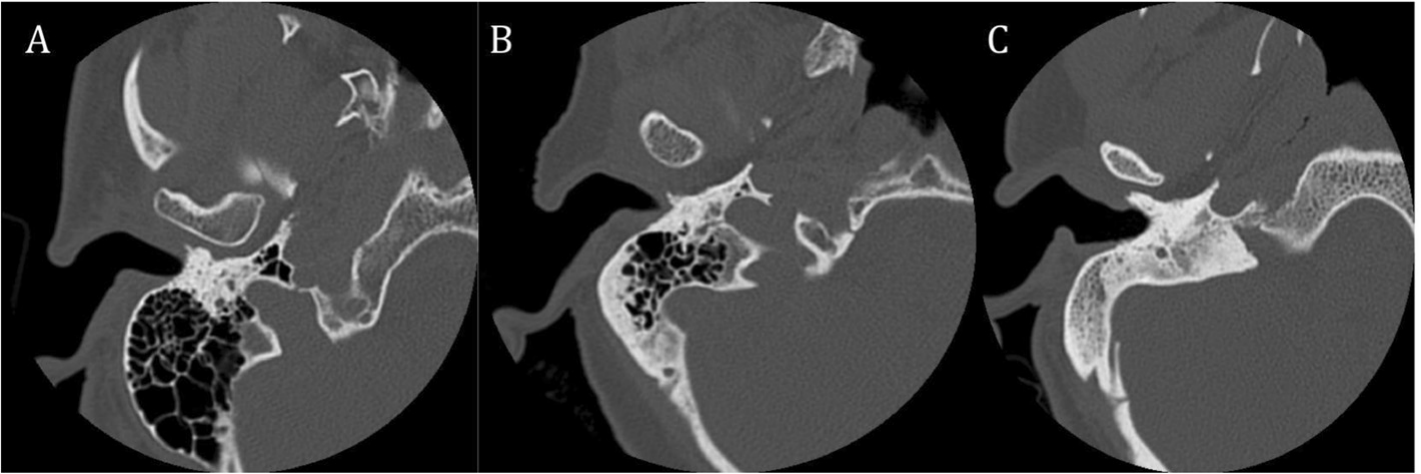
Representative axial images of pre-operative high-resolution temporal bone CT scan showing mastoid that is (**a**) well aerated, (**b**) moderately aerated, and (**c**) poorly aerated

With respect to mastoid aeration, the mastoid on the ipsilateral side of cochlear implant was examined on axial images. The degree of its aeration was categorized as being either “well aerated”, “moderately aerated”, or “poorly aerated” (Fig. 1a-c).

**Fig. 2:**
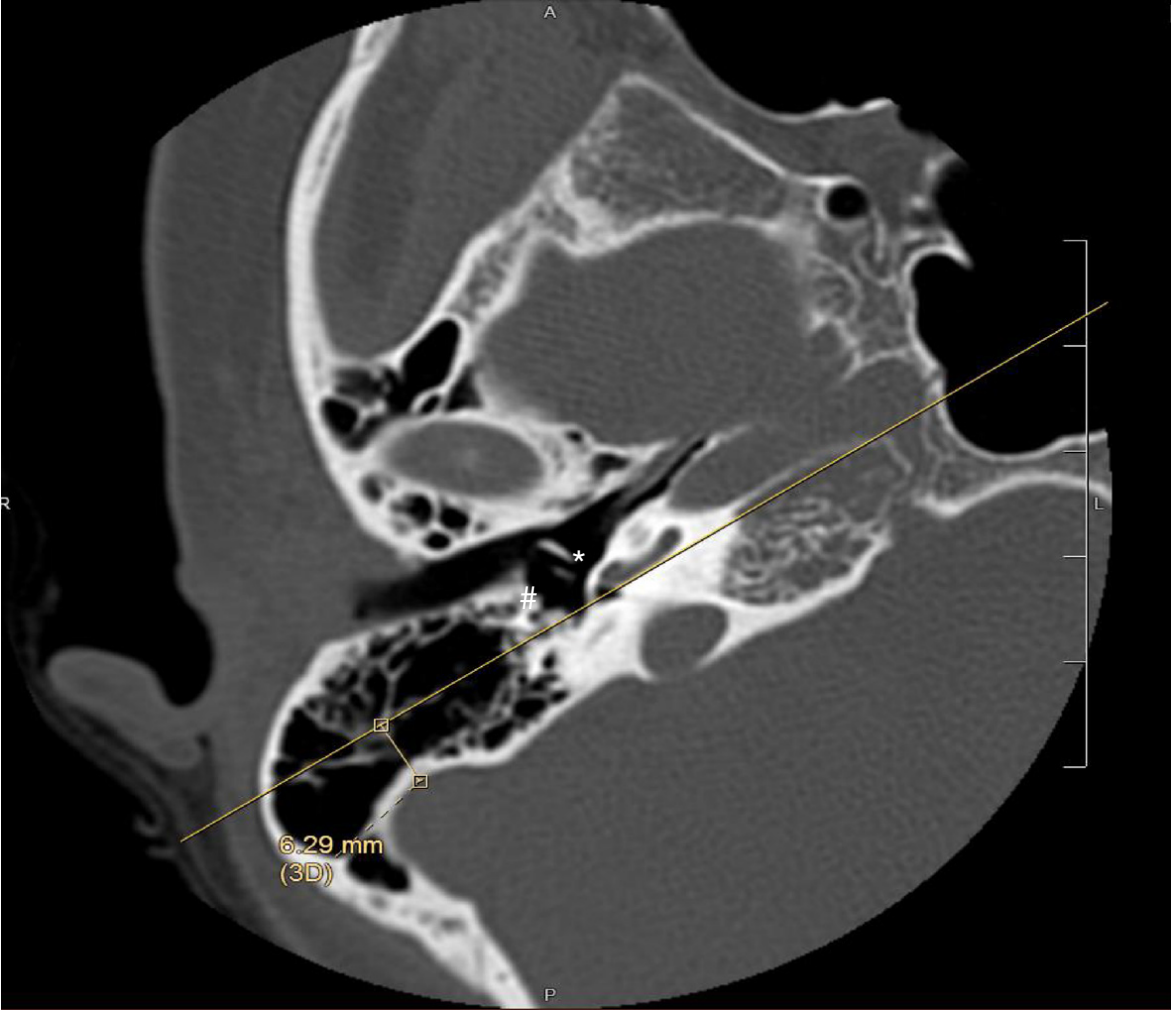
Representative axial image of pre-operative high-resolution temporal bone CT scan illustrating the distance measured from the line drawn through round window (*) and facial nerve (#) to the anterior aspect of the sigmoid sinus

Location of the sigmoid sinus was measured on the same axial images as above. A straight line was first drawn through the mid-portion of the round window and the facial nerve, thus bisecting these landmarks. Subsequently, a perpendicular line from this axis to the most anterior aspect of the sigmoid sinus was drawn and measured (in mm) (Fig. 2).

**Fig. 3:**
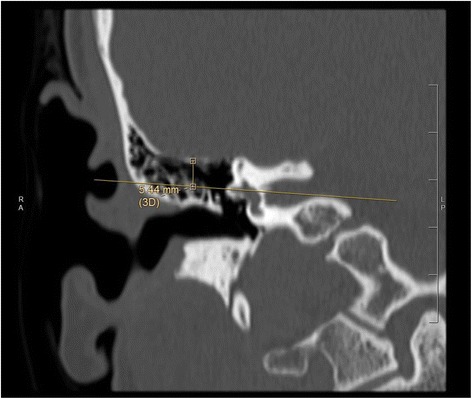
Representative coronal image of pre-operative high-resolution temporal bone CT scan illustrating the distance measured from the line drawn through horizontal semicircular canal to the tegmen

To determine the height of the tegmen, a straight line was drawn through the axis of the horizontal semicircular canal on a coronal image. Subsequently, a perpendicular line from this axis to the lowest level of tegmen was drawn and measured (in mm) (Fig. 3). These three parameters were then compared to the intraoperative scoring of difficulty associated with the cortical mastoidectomy.

**Fig. 4:**
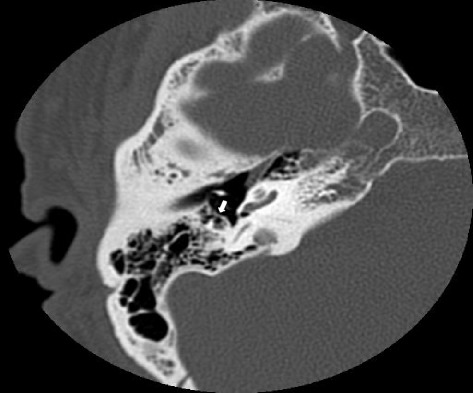
Representative axial image of pre-operative high-resolution temporal bone CT scan illustrating an air cell (arrow) anterior to facial nerve

In addition, using the axial images, presence or absence of air cells around the facial recess was assessed (Fig. 4). This was correlated to the intraoperative scores of difficulties with access to facial recess.

**Fig. 5:**
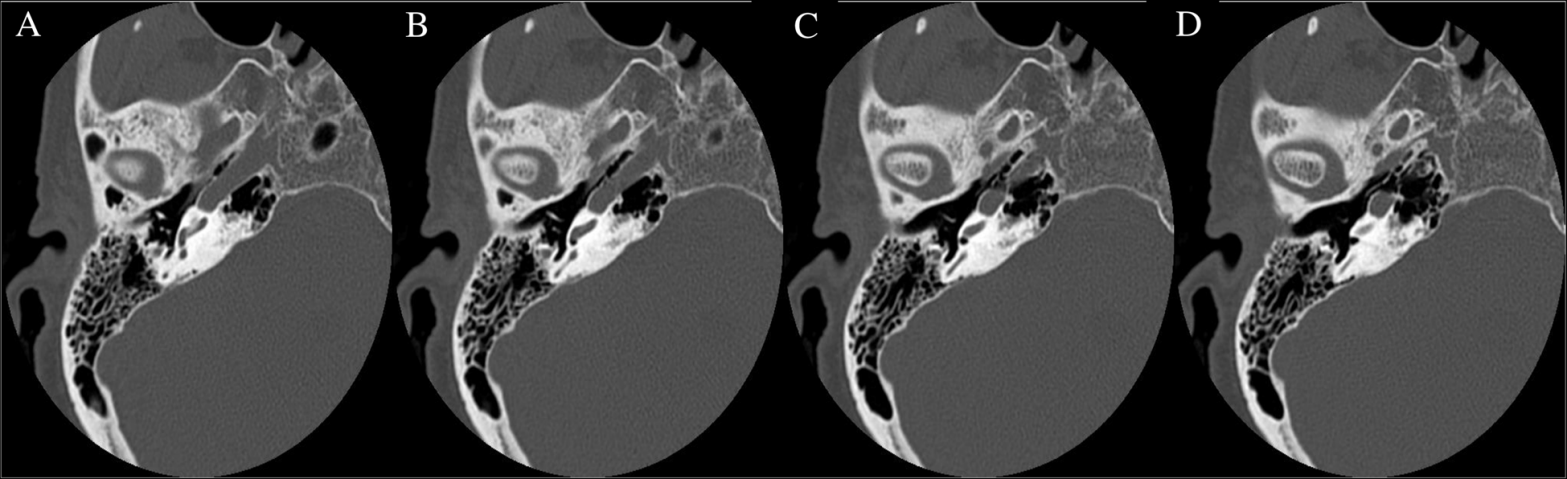
Representative axial images of pre-operative high-resolution temporal bone CT scan illustrating bony overhang around round window in four consecutive slices from superior to inferior (**a** to **d**)

Finally, the degree of round window bony overhang was measured by assessing four consecutive axial cuts, beginning with the most superior cut showing the round window membrane and proceeding inferiorly (Fig. 5). The number of cuts showing full thickness bony overhang around the round window was counted out of four. For instance, if there were two slices showing full thickness bony overhang, it was measured as 2/4 or 0.5. This variable was then compared to the intraoperative scoring of difficulty associated with round window access.

### Statistical analysis

A chi-square test of independence was used to find a potential relationship between difficulty with mastoidectomy and mastoid aeration, as well as a potential relationship between difficulty with accessing facial recess and presence/absence of facial recess air cell.

One-way analysis of variance (ANOVA) and student’s *t*-test were used to compare the location of the sigmoid and the level of the tegmen between the three groups representing degrees of difficulties with mastoidectomy (i.e. “Easy”, “Moderate”, “Difficult”). In addition, one-way ANOVA and student’s *t*-test were used to compare level of round window bony overhang between the three groups of surgical difficulty.

## Results

### Demographics

The average age of subjects was 58 (range: from 21 to 84). There were 29 males and 28 females in the study. Twenty-eight patients underwent right cochlear implantation and 29 patients underwent left cochlear implantation.

### Mastoidectomy

A chi-square test of independence was utilized to assess whether the difficulty encountered during the cortical mastoidectomy is related to the degree of mastoid aeration as assessed on the pre-operative temporal bone CT scan. The analysis revealed a chi-square value of 26.7 that is significant at the *p*-value of <0.001, demonstrating that lower degree of mastoid aeration is associated with higher level of difficulty during the cortical mastoidectomy.

The distance between the straight line drawn through the round window and the facial nerve, and the sigmoid sinus on the pre-operative CT scan provides an indication of how anterior the sigmoid sinus is. The mean distance was compared between the three groups, which corresponded to the levels of difficulty with the cortical mastoidectomy (“Easy” =7.11 ± 0.34, “Moderate” =6.39 ± 1.74, “Difficult” =5.15 ± 1.74; mean SEM). One-way ANOVA revealed that the difference in mean distance of sigmoid sinus between the three groups is not statistically significant. When student’s *t*-test was performed between the groups separately, none of the comparisons was statistically significant.

**Fig. 6:**
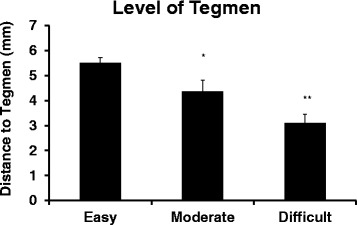
Mean distance between the axis of the horizontal semicircular canal and the lowest level of the tegmen on coronal CT images among the three groups corresponding to the degree of difficulty associated with mastoidectomy. *: *p* = 0.05 vs “Easy”, **: *p* < 0.01 vs “Easy”

The height of the tegmen on pre-operative CT scan was analyzed in similar manner. The mean distance of tegmen from the axis of horizontal semicircular canal was significantly different between the three groups based on one-way ANOVA (“Easy” = 5.50 ± 0.22, “Moderate” = 4.36 ± 0.46, “Difficult” = 3.11 ± 0.35; *p* < 0.01). The mean distance for the “Moderate” group was significantly lower than the “Easy” group (*p* = 0.05), while the mean distance for the “Difficult” group tended to be lower than the “Moderate” group, although the difference was not statistically significant (*p* = 0.06) (Fig. 6).

### Facial recess access

A chi-square test of independence was used to determine whether presence or absence of an air cell around the facial recess on pre-operative CT scan is a predictor for the degree of difficulty with accessing facial recess. The result shows that the presence or absence of an air cell around the facial nerve is significantly related to the degree of difficulty with accessing the facial recess (*p* = 0.05), with the presence of an air cell being associated with an ‘easier’ rating of facial recess.

### Round window access

The degree of round window bony overhang was compared between the three groups corresponding to levels of difficulty associated with round window access using one-way ANOVA. There was an overall statistical difference among the groups (*p* = 0.02). However, individual student’s t-tests did not show statistically significant differences for all paired comparisons.

## Discussion

The objective of this study was to determine if there are potential predictors of difficulties associated with the key surgical steps during cochlear implantation based on an analysis of the pre-operative temporal bone CT scan. These well-established steps include cortical mastoidectomy, facial recess, and round window exposure, which are not only important for successful insertion of the cochlear implantation electrode through the round window, but in fact are related since each subsequent step is dependent upon the previous steps being done properly and adequately. This study demonstrates that the difficulty associated with the cortical mastoidectomy is related to the degree of mastoid aeration and the height of the tegmen, and that the difficulty associated with facial recess is related to the presence/absence of an air cell around facial nerve.

Pre-operative CT scans are currently the standard of care for adult patients undergoing cochlear implantation. These images are routinely reviewed by the surgeon prior to surgery and a mental checklist is often performed to ensure that there are no anatomical obstructions to implant insertion. If there is any anatomical variation, the surgeon is not only better prepared intraoperatively, but a frank discussion can be undertaken with the patient pre-operatively about alternative surgical steps, such as transcanal insertions or removal of the posterior canal wall.

However, there can be a high degree of variability in how the pre-operative CT scan is analyzed by the individual surgeon. Our goal was to formalize and standardize various radiological markers which can be used to prognosticate the potential challenges the surgeon may encounter during surgery. This planning is similar to obtaining a MRI to assess cochlear duct patency in post-meningitis patients who suffer profound sensorineural hearing loss and are requiring urgent cochlear implantation.

Our finding that decreased mastoid aeration and lower level of tegmen are associated with a greater level of perceived difficulty associated with cortical mastoidectomy is not surprising. In a sclerotic or small mastoid, identifying important landmarks required for subsequent steps, such as the identification of the lateral semicircular canal and incus body, is naturally more difficult. These are well established otologic landmarks that help the surgeon identify the location of the facial nerve and mastoid antrum. Without this identification, the risks of iatrogenic injury to the facial nerve and inner ear are significantly increased. Similarly, a low tegmen can also slow down the surgeon and make the exposure of the mastoid antrum during cortical mastoidectomy more challenging. However, our findings do not rule out other anatomical or radiological markers that may contribute to technical challenges associated with mastoidectomy.

Interestingly, there was no significant association between the location of sigmoid sinus (in other words, how anterior its location is) and the degree of difficulty with mastoidectomy. It may be that even in patients whose mastoidectomy was “difficult”, the sigmoid sinus was sufficiently away from the surgical field. This may also be related to the type of surgery. In cochlear implantation, most of the surgical dissection is located anterior to the sigmoid sinus within a limited mastoidectomy, as opposed to a wide, ‘saucerized’ cavity required for cholesteatoma or acoustic neuroma surgery.

Our results also show that the presence of an air cell around the facial recess on the preoperative CT scan is associated with lower degree of difficulty with facial recess access. Again, this is not surprising, as surgeons welcome the presence of an air cell in the facial recess, which is then used to confirm opening into the middle ear and guides rest of the facial recess enlargement.

During round window exposure, a thick bony overhang can pose problems for the surgeon. A thick bony overhang often precludes the true location and orientation of the round window. This overhang must be drilled away to expose the round window and to allow smooth insertion of the electrode into the perilymphatic space of the cochlea unhindered by bony obstructions. A thick bony overhang was assessed radiologically by assessing four consecutive axial cuts of the pre-operative CT scan. A thicker round window bony overhang was not associated with greater difficulty in accessing round window. It is likely that the orientation and size of the round window (i.e. how posterior it is), rather than thickness of the bony overhang, better predicts the difficulty with round window access, but this was not tested in our radiological markers. Most of the patients who received a cochlear implant also had little evidence of chronic mastoid disease, which made identification of the overhang easier.

There are several limitations with our study. The descriptors for difficulty associated with different steps of the cochlear implant are subjective and what is deemed as “difficult” vs “moderate”, for instance, may vary widely between surgeons. Furthermore, only one investigator analyzed the CT images and therefore inter-observer reliability or variability is not known. This will be addressed in the future studies.

## Conclusion

The results of our study show that 1) aeration of the mastoid and height of the tegmen may help predict the degree of difficulty with cortical mastoidectomy and 2) the presence of air cell around the facial recess may be a predictor of an easier facial recess.

These radiological parameters are relatively easy and quick to assess on readily available pre-operative temporal bone CT scan. They can form a pre-operative checklist that provides a formalized approach for the surgeons and, in particular surgical trainees, predict and, thus prepare for, potentially challenging cochlear implant cases.
